# Microbial community spatial structures in Luzhou-flavored liquor pit muds with different brewing materials

**DOI:** 10.7717/peerj.12987

**Published:** 2022-03-08

**Authors:** Jinjin Li, Hongzhao Sun, Qian Wang, Yunfei Cai, Zhu Shi, Jianlei Jia, Lei Zheng, Ru Jiang, Lingmei Gao

**Affiliations:** 1 School of Life Sciences, Qilu Normal University, Jinan, Shandong, China; 2 Jinan High-Tech Zone Experimental Middle School, Jinan, Shandong, China; 3 Shandong Baimai Spring Wine Co., Ltd, Jinan, Shandong, China; 4 Shandong Yinlu Food Co., Ltd, Jinan, Shandong, China

**Keywords:** Pit mud, Microbial community, Spatial distribution, Luzhou-flavor Baijiu, High-throughput sequencing

## Abstract

**Background:**

Although studies have shown that Bacteroidetes, Clostridiales, and Lactobacillales are the main components of the microbial community in pit mud during the brewing of Luzhou-flavored liquor, little is known about the effect of brewing materials on spatial structures of this microbiome.

**Methods:**

High-throughput sequencing of the V4–V5 region of prokaryotic 16S rRNA gene was performed to analyze the microbial community diversity and spatial heterogeneity in Luzhou-flavored liquor pit muds with different brewing ingredients. The structural characteristics and heterogeneous spatial distribution of the pit mud microbial communities were examined using bioinformatics and multivariate statistical analysis methods.

**Results:**

Our results showed that Euryarchaeota, Actinobacteria, Bacteroidetes, Chlorobi, Chloroflexi, Firmicutes, Proteobacteria, Synergistetes, Tenericutes, and WWE1 were the dominant phyla in the pit mud microbiome. The Shannon and Simpson indices of the pit mud microbiome with three grains (M3G) in the upper layer were significantly lower than those in middle layer and bottom, whereas those of the pit mud microbiome with five grains (M5G) in bottom were significantly lower than those in middle layer (*p* < 0.05). There were significant differences in the microbial community compositions between the pit muds with different brewing ingredients and locations in the same pit (*p* < 0.05). T78 of Anaerolinaceae, *Butyrivibrio*, *Dehalobacter_Syntrophobotulus*, *Desulfosporosinus*, *Asteroleplasma*, and vadinCA02 of Synergistaceae were significantly enriched in M3G, whereas *Prevotella*, *Vagococcus*, *Caldicoprobacter*, *Butyrivibrio*, *Coprococcus*, *Dorea*, *Sporanaerobacter*, *Tepidimicrobium*, *TissierellaSoehngenia*, RFN20 of Erysipelotrichaceae, *Sutterella*, 125ds10 of Alteromonadales, *Vibrio*, and *Sphaerochaeta* were significantly enriched in M5G. This study provides a theoretical basis for exploring the influence of brewing ingredients in pit muds on the production of Luzhou-flavored liquor and the specific influence of pit mud microorganisms in different locations on liquor production.

## Introduction

Chinese liquor, also called Baijiu, has a long history in China, and various flavors, such as Maotai, Luzhou, Fen, and Phoenix, have been developed (Zheng & Han, 2016; Zheng et al., 2016). Luzhou-flavored Baijiu, manufactured from fermented grains (such as rice, sorghum wheat, peas, and millet) in a soil cellar, has the highest annual production rate of all flavors (Ding et al., 2014; Sakandar et al., 2020). After traditional solid-state fermentation, distillation, aging, and blending, the liquor is formed (Fig. S1; Zhang et al., 2009; Liu & Li, 2014). The formation of unique components and flavor characteristics of Luzhou-flavored Baijiu depends on the materials and energy metabolism of the microbial communities in the solid, liquid, and gas phases of production (Zhang et al., 2005). Raw materials are fermented by pit microorganisms and produce alcohols, aldehydes, acids, esters, ketones, lactones, acetals, pyrazines, furans, aromatic compounds, and sulfides, which determine the quality and unique flavor of the liquor (Kang & Xu, 2012). Among these compounds, ethanol and ethyl caproate are the main determinants of flavor (Liu et al., 1995). Pit mud carries microorganisms that produce these compounds, therefore, it is directly related to the main components of Luzhou-flavored Baijiu and plays a key role in the brewing process (Tao et al., 2014a).

Prokaryotic community diversity in brewing pit mud has been shown as positively correlated with pit age (Deng et al., 2012; Tao et al., 2014a), depth (Luo et al., 2017), and Baijiu quality (Hu et al., 2016). Studies have shown that as the pit age and Baijiu quality increase, the relative abundance of Firmicutes decreases, whereas that of Bacteroidetes and Euryarchaeota increases (Tao et al., 2014b; Hu et al., 2016). Spatially, a study found that the relative abundance of Bacteroidetes in new pit mud was higher than that in new pit walls (Luo et al., 2017). However, although studies have shown that Bacteroidetes, Clostridiales, and Lactobacillales are the main components of the microbial community in pit mud (Zhang et al., 2015), little is known about the spatial heterogeneity of the microbial community in this particular environment.

In addition to pit characteristics, the composition of brewing raw materials affects the flavor and quality of Baijiu. For instance, in a study comparing different grain sources on Baijiu flavor, Chen et al. (2008) found that the flavor of Bajiu made with sorghum was fragrant and sweet. The addition of corn as an auxiliary material increased the sweetness, and an 8% addition of peas made the flavor mild. However, the use of pea alone resulted in poor aroma and quality. Fang et al. (2021) reported differences in flavor components, such as ethyl acetate, ethyl butyrate, and ethyl valerate, in Baijiu made with millet compared to that made with five other raw materials. While compounds and liquor qualities have been investigated, information regarding the impact of raw material on the spatial heterogeneity of the pit microbial community is lacking.

The aim of this study was to investigate the effect of brewing material on pit mud microbial community structure during the production of Luzhou-flavored Baijiu. Considering the impact of different raw materials on the flavor and quality of Baijiu, we hypothesized that different combinations of brewing raw materials would change the microbial community structure and spatial distribution in the pit mud. To test this hypothesis, we analyzed the microbial community structures in pit mud used for brewing Baijiu with different grain mixtures using high-throughput sequencing technology. The spatial microbial distribution and community diversity was also examined in different parts of the pit.

## Materials and Methods

### Sample collection

**Figure 1:**
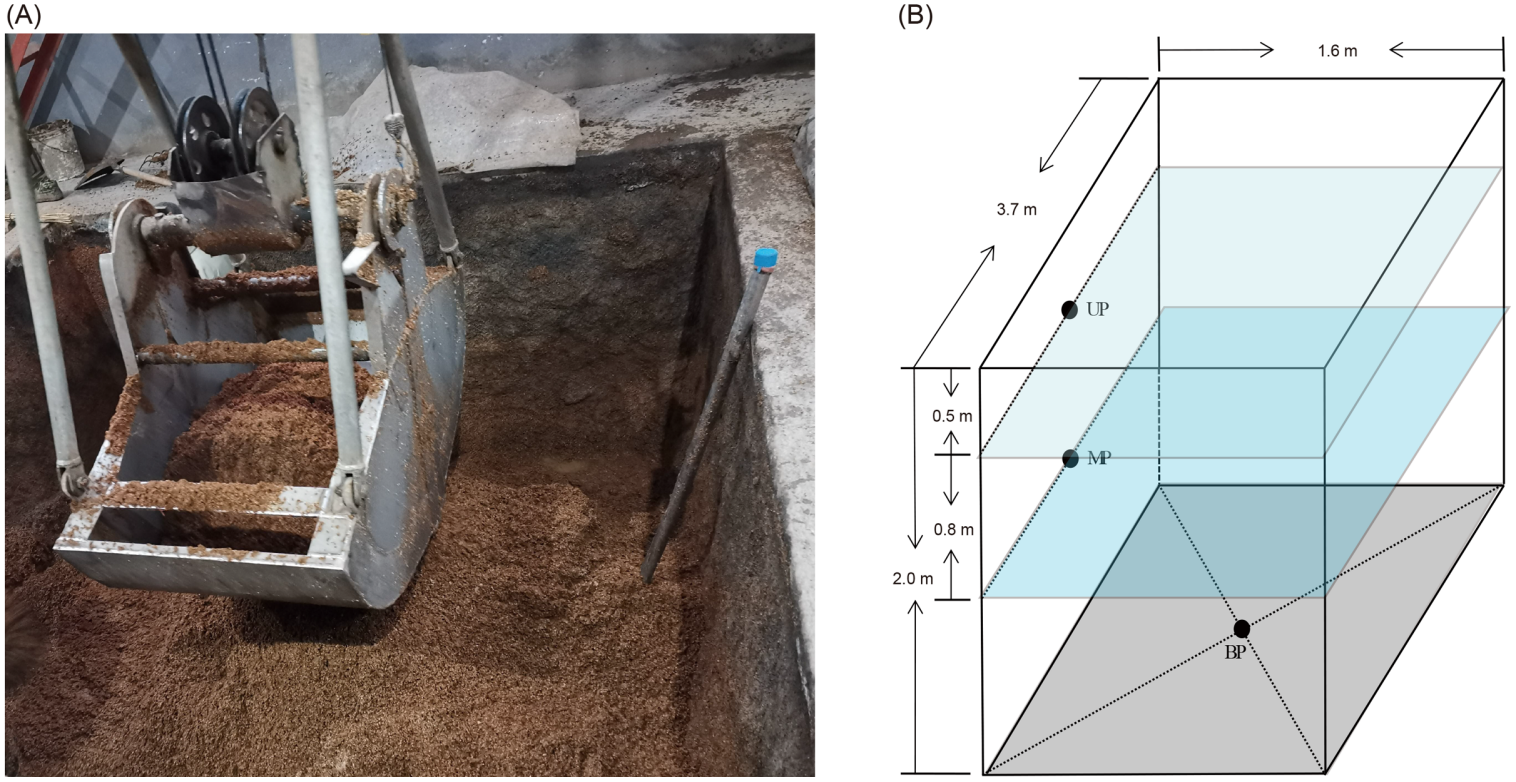
Luzhou Baijiu brewing cellar with raw grains (A) and distribution of the sampling sites (B). The black points in the panel (B) indicate the sampling layers of the pit muds. Photo credit: Lei Zheng.

Pit mud samples were collected from three- or five-grain Luzhou-flavored Baijiu brewing pits at Shandong Baimai Spring Wine Co., Ltd., China (Fig. 1A) after 60 days of fermentation. The two pits were of the same age and used the same fermentation starter (Daqu). The three-grain Baijiu (M3G) used sorghum (50% by weight), rice (25% by weight), and corn (25% by weight). The five-grain Baijiu (M5G) used sorghum (36% by weight), rice (22% by weight), corn (8% by weight), wheat (16% by weight), and glutinous rice (18% by weight). The length, width, and height of the liquor cellars were 3.7, 1.6, and 2.0 m, respectively (Fig. 1B). The samples were collected from the upper layer (0.5 m from the cellar surface, UP), the middle layer (1.3 m from the cellar surface, MP), and the bottom (BP) of the cellars (Fig. 1B). The pit mud was randomly collected from three points in the same layer and mixed as a sample. Five samples were collected from each layer. Five grams of pit mud were collected from each sampling site, transferred into 50 mL sterile Eppendorf tubes, quickly placed in an icebox, and transported to the laboratory for storage at –20 °C before DNA extraction.

### DNA extraction and high-throughput sequencing

Approximately 1 g of pit mud from each sample was used for DNA extraction. The total genomic DNA of the pit mud microbial community was extracted using a Power Soil DNA Extraction Kit (QIAGEN, Germany). DNA concentration was determined using a Nanodrop 2000 spectrophotometer and diluted to 10 ng/µL for polymerase chain reaction amplification. The V4–V5 hypervariable region of the prokaryotic 16S rRNA gene isolated from the samples was amplified using prokaryotic universal primers 515F (5′-GTGYCAGCMGCCGCGGTA-3′) and 909R (5′-CCCCGYCAATTCMTTTRAGT-3′) (Tamaki et al., 2011). The 5′ end of primer 515F was connected to a 12 nucleotide barcode sample-specific sequence for subsequent sample division. Sequencing was conducted using the Illumina HiSeq platform at Guangdong Meilikang Bio-Science Ltd., China (Ni et al., 2021).

The raw reads were merged using FLASH 1.2.8 and filtered using QIIME 1.9.0 (Caporaso et al., 2010) to remove sequences with one or more of the following attributes: mismatch with primers, length of less than 300 bp, presence of ambiguous base “N,” or average base quality of less than 30 (Ni et al., 2017). Chimeric sequences were detected and removed from the remaining sequences using UCHIME (Edgar et al., 2011). The remaining high-quality sequences were then clustered into operational taxonomic units (OTUs) based on 97% sequence similarity using USEARCH (Edgar, 2013). Alpha diversity indexes and weighted UniFrac distances were calculated, and principal coordinates analysis (PCoA) based on weighted UniFrac distances was conducted using QIIME 1.9.0. The OTUs were annotated to taxa using the Ribosomal Database Project Classifier (Wang et al., 2007) with the Greengenes gg_13_8 dataset (http://qiime.org/home_static/dataFiles.html).

### Data analysis

Data were expressed as the mean ± standard deviation. The Kruskal-Wallis rank sum test with the Tukey–Kramer post-hoc test was performed using R 3.5.1 (R Core Team, 2013). Partial redundancy analysis (RDA) and permutational multivariate analysis of variance (PERMANOVA) were performed using the ‘vegan’ package in R 3.5.1 (Anderson, 2001). The Kruskal–Wallis H test was performed using STAMP software to screen for OTUs with significant differences among different groups (Parks et al., 2014). A heatmap was drawn using the pheatmap package in R 3.5.1, and different taxa among groups were detected based on linear discriminant analysis effect size (LEfSe) (Segata et al., 2011). Statistical significance was set at *p* < 0.05.

## Results

**Figure 2:**
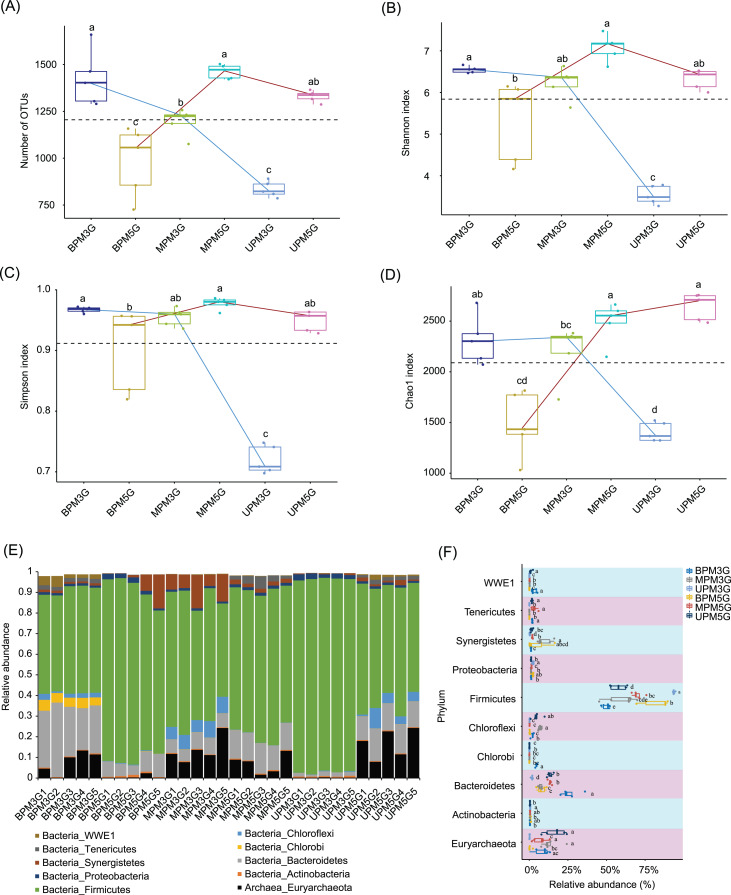
Changes in alpha diversity indexes (A–D) and phylum compositions (E–F) of pit mud microbial communities. (A) Number of observed operational taxonomic units (OTUs), (B) Shannon index, (C) Simpson index, (D) Chao1 index, (E) phylum compositions, and (F) differences in each phylum between different groups. Different lowercase letters in the upper (A–D) or right (E) part of the boxes indicate significant differences between groups.

To eliminate the effect of different sequencing depths on the results, 21,698 high-quality sequences were randomly selected from each sample for subsequent analysis. In total, 10,898 OTUs were detected in the samples. PCoA based on weighted UniFrac distances showed that there were significant differences between M5G and M3G in the same pit layer (PERMANOVA, *n* = 5, *df* = 1, *F* = 49.45, *p* = 0.014 for UP, *n* = 5, *df* = 1, *F* = 9.93, *p* = 0.015 for MP, *n* = 5, *df* = 1, *F* = 12.04, *p* = 0.008), and between the microbial communities in different layers of the same cell (PERMANOVA, *n* = 5, *df* = 2, *F* = 9.54, *p* < 0.001 for M5G; n = *5*, *df* = 2, *F* = 43.75, *p* < 0.001 for M3G; *n* = 5, *df* = 2, *F* = 12.04, *p* = 0.008; Fig. S2). Partial RDA results showed that the contributions of pit location and brewing materials to the microbial community differences were 30.12% and 4.17%, respectively (Fig. S3). The alpha diversity analysis results showed that the OTU numbers of M3G decreased from BP to UP (Kruskal-Wallis rank sum test, *χ*^2^ = 12.5, *df* = 2, *p* = 0.002). In contrast, the OTU numbers of M5G in BP were significantly lower than those in MP and UP (Kruskal-Wallis rank sum test, *χ*^2^ = 12.5, *df* = 2, *p* = 0.002; Fig. 2A). The Shannon indices of BPM3G, BPM5G, MPM3G, MPM5G, UPM3G, and UPM5G were 6.55 ± 0.03, 5.32 ± 0.43, 6.23 ± 0.17, 7.08 ± 0.14, 3.53 ± 0.10, and 6.32 ± 0.11, respectively (Fig. 2B). The Shannon and Simpson indices of M3G in UP were significantly lower than those in MP and BP (Kruskal-Wallis rank sum test, *χ*^2^ = 10.82, *df* = 2, *p* = 0.004 for Shannon index; *χ*^2^ = 10.22, *df* = 2, *p* = 0.006 for Simpson index), whereas those of M5G in BP were significantly lower than those in MP (Kruskal-Wallis rank sum test, *χ*^2^ = 11.18, *df* = 2, *p* = 0.004 for Shannon index; *χ*^2^ = 9.06, *df* = 2, *p* = 0.011 for Simpson index; Figs. 2B and 2C). The Chao1 index of M3G in UP was significantly lower than that in the other layers (Kruskal-Wallis rank sum test, *χ*^2^ = 9.38, *df* = 2, *p* = 0.009), whereas that of M5G in BP was significantly lower than that in the other layers (Kruskal-Wallis rank sum test, *χ*^2^ = 10.22, *df* = 2, *p* = 0.006; Fig. 2D). These results implied that there were significant differences in the microbial community structure and the spatial distribution of alpha diversity between M3G and M5G.

Except for a few that could not be classified into any phylum, all sequences were classified into 56 phyla (including two archaea and 54 bacterial phyla), of which Firmicutes, Bacteroidetes, Euryarchaeota, Actinobacteria, Synergistetes, Chloroflexi, Chlorobi, Proteobacteria, Tenericutes, and WWE1 were the most abundant (Fig. 2E). The relative abundance of Firmicutes in M3G decreased gradually from UP (93.65 ± 0.28%) to BP (49.96 ± 1.14%), whereas the relative abundance of Bacteroidetes increased gradually from UP (1.76 ± 0.23%) to BP (26.24 ± 2.72%). Synergistetes (Kruskal-Wallis rank-sum test, *χ*^2^ = 12.5, *df* = 2, *p* = 0.002) and Euryarchaeota (Kruskal-Wallis rank-sum test, *χ*^2^ = 8.54, *df* = 2, *p* = 0.014) were significantly enriched in both pit mud microbial communities in MP (Fig. 2F). In contrast to M3G, the proportion of Firmicutes in M5G gradually increased from UP (57.76 ± 2.32%) to BP (82.15 ± 4.09%), whereas the proportion of Euryarchaeota gradually decreased from UP (16.85 ± 3.16%) to BP (0.57 ± 0.44%; Fig. 2F).

**Figure 3:**
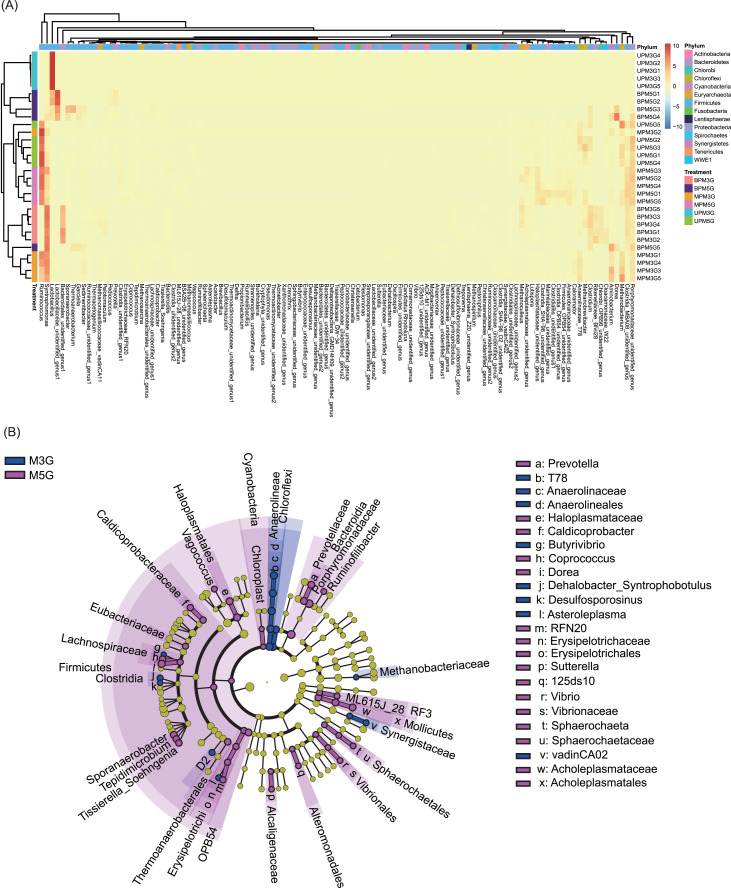
Effects of different brewing grains and cellar spatial locations on the composition of pit mud microbial communities. (A) Heatmap of dominant bacterial genera in the pit mud microbial communities. (B) Cladogram plot showing the linear discriminant analysis effect size results.

At the genus level, 131 dominant genera (relative abundance >1% in at least one sample) were detected in the 30 samples. Based on these genera, different pit mud samples were clustered into different groups according to their sampling sites (with the exception of UPM5G5, MPM3G2, and BMP5G5; Fig. 3A). Although the microbial communities in BP and MP of M3G and in MP and UP of M5G were clustered together, the microbial communities in UP of M3G BP of M5G were clustered together (Fig. 3A). The LEfSe results showed that *Butyrivibrio*, *Dehalobacter_Syntrophobotulus*, *Desulfosporosinus*, *Asteroleplasma*, T78 of Anaerolinaceae, and vadinCA02 of Synergistaceae were significantly enriched in M3G, whereas *Prevotella*, *Vagococcus*, *Caldicoprobacter*, *Butyrivibrio*, *Coprococcus*, *Dorea*, *Sporanaerobacter*, *Tepidimicrobium*, *Tissierella_Soehngenia*, RFN20 of Erysipelotrichaceae, *Sutterella*, 125ds10 of Alteromonadales, *Vibrio*, and *Sphaerochaeta* were significantly enriched in M5G (LEfSe, *n* = 5, LDA score (log10) > 2; Fig. 3B and Fig. S4). Moreover, the relative abundances of most of the dominant genera were significantly different between the different layers (LEfSe, *n* = 5, LDA score (log10) > 2; Figs. S5 and S6). These results indicated that the composition of the liquor-making materials significantly affected the composition and spatial distribution of the mud microbial community in Luzhou-flavored Baijiu.

## Discussion

There are significant differences in the microbial community structure of pit mud among different brewing enterprises (Tang et al., 2012; Zheng et al., 2013; Ding et al., 2015). The diversity of the microbial composition in pit mud may be the main driver of quality and flavor differences in Luzhou-flavored Baijiu across regions and enterprises in China. Tao et al. (2014b) reported that Firmicutes (66.8%), Bacteroidetes (16.0%), Euryarchaeota (9.0%), Actinobacteria (1.8%), and Chloroflexi (1.0%) were the dominant phyla in the microbial community of pit mud in Luzhou-flavored Baijiu. In agreement with these results, these bacterial phyla were also detected in the pit mud microbial communities in our study. However, we found Chlorobi, Proteobacteria, Synergistetes, Tenericutes, and WWE1 to be the dominant phyla. This could be explained by various factors, such as pit age or raw brewing material.

The aromatic substances caproic acid, butyric acid, octanoic acid, and their corresponding ethyl esters are precursors of Luzhou-flavored Baijiu. Previous studies have shown that *Clostridium*, *Lactobacillus*, and unidentified genera of Clostridiaceae and Anaerobrancaceae are the core microorganisms of the pit mud of Luzhou-flavored Baijiu (Tao et al., 2014b) with ethyl hexanoate, an acid derivative, as a main flavor component (Liu et al., 1995; He et al., 2017). An abundance of *Lactobacillus* has been reported to be negatively correlated with the formation of caproic acid, whereas that of the other three genera is positively correlated with caproic acid production (Tao et al., 2014b; Liu et al., 2019). These core microorganisms were also found in our pit mud samples, indicating that they play an important role in the flavor composition of Luzhou-flavored Baijiu. *Aminobacteria* can ferment a variety of amino acids and further expand the types of substrate amino acids *via* interactions with other bacteria (Baena et al., 1998). Previous studies have shown that *Sedimentibacter* is a major prokaryotic microorganism present in pit mud and ferments amino acids to ethanol, acetic acid, and butyric acid (Obst et al., 2005), as well as producing sulfide from specific substrates (Tskii et al., 2007). Our results showed that *Aminobacterium* and *Sedimentibacter* were only detected as dominant genera in the MP of M3G. *Syntrophomonas* usually coexists with methanogenic bacteria and relieves hydrogen inhibition *via* interspecific “hydrogen transfer” to effectively degrade long-chain hydrolytic fatty acids into short-chain fatty acids, making the reaction conducive to the production of caproic acid (Madigan et al., 2012). However, our results showed that *Syntrophomonas* was only detected as a dominant genus in the BP of M3G. Methanogenic bacteria, such as unidentified genera of Methanomicrobiales, Methanomicrobia, Methanomicrobiaceae, and *Methanoculleus*, were also detected in the BP of M3G. Qian et al. (2021) reported that although lactic acid accumulated in jiupei, butyric and caproic acids were mainly produced by microbes inhabiting pit mud. Therefore, the particular light aromatic flavor or taste of M3G might be related to the specific dominant genera in the cellar. However, the specific mechanism of this phenomenon requires further investigation.

As lactic acid-producing bacteria inhibit caproic acid production (Yao et al., 2010), the relative abundance of *Lactobacillus* is negatively correlated with the production of caproic acid (Tao et al., 2014a). Therefore, the content of *Lactobacillus* in a degraded pit mud is high (Liu et al., 2019). Our results showed that *Lactobacillus* was only detected as the dominant bacterium in the UP samples of M3G. This implied that there was a degradation risk in the pit, and the degradation risk of M3G was higher than that of M5G. However, this must be confirmed experimentally.

Raw materials to produce Baijiu are of paramount importance because they are the source of flavor compounds in the final product (Du et al., 2019). Although the composition of raw brewing materials affects the flavor and quality of Baijiu (Chen et al., 2008; Fang et al., 2021), the effect of raw materials on the microbial community structure in pit mud has not been deeply investigated. Our results showed that the composition of raw materials significantly affected the composition and spatial distribution of the microbial community in the Luzhou-flavored Baijiu pit muds. There are two plausible reasons for these results. First, different raw materials contain different nutrients, which affect the composition of the microbial community in the pit mud. Secondly, variation in the physical properties of raw materials, especially viscosity, can affect the transmission of chemical components and microbial metabolism in pit mud microorganisms.

A previous study showed spatial differences in microbial abundance and community composition in Baijiu cellar muds at different locations in a single pit, but these differences were not significant (Hu et al., 2020). Our results indicated that the microbial community structure of the Baijiu cellar mud was significantly affected by the brewing materials, and there was significant spatial heterogeneity in the microbial community structure in the pit mud.

Genera belonging to Clostridia, Methanobacteria, Methanomicrobia, and Synergistia have been shown to play pivotal roles in the metabolic network of pit mud microbial communities (Hu et al., 2016). Our results showed that an unidentified genus of Clostridiaceae was the dominant genus in the BP of M3G. An unidentified genus, Methanobacteria, Methanobacteriales, and *Methanobrevibacter* were detected only in the UP of M5G. *Methanobacterium* was only detected in the MP of M3G, and an unidentified genus, Methanomicrobia, Methanomicrobiales, Methanomicrobiaceae, and *Methanoculleus* were only detected as dominant genera in the BP of M3G. Moreover, an unidentified genus, Synergistia, Synergistetes, and Synergistales, were only detected as dominant genera in the MP of M3G. *Clostridium* was only detected as a dominant bacterium in the BP of M3G and M5G. These results implied that there was spatial heterogeneity in the microbial composition of the pit mud at different locations in both M3G and M5G cells.

Previous studies have shown that the Shannon index values of high-quality and aged pit mud microbial communities were 5.83 and 6.45, respectively (Hu et al., 2016; Luo et al., 2017). Although these results suggest that the risk of pit mud aging increases with the Shannon index, our results showed that the Shannon index of UPM3G was the lowest of all samples (3.53 ± 0.10), followed by BPM5G (5.32 ± 0.43) and MPM3G (6.23 ± 0.17). The Shannon index of MPM5G was the highest (7.08 ± 0.14). These results suggest that the Shannon index of the pit mud microbial community is probably more affected by liquor-making materials and location in the cellar. Moreover, a previous study showed that the microbial community in pit mud increased with sampling depth in degraded pits, implying that the degradation process occurred from bottom to top (Hu et al., 2021). Our results showed that the OTU number and Chao1 index of microbial communities in the UP of M3G and BP of M5G were significantly lower than those in the other two layers of the same pit, and the Shannon and Simpson indices of UP of M3G were significantly lower than those of the other two layers of the same pit. Although the Shannon and Simpson indices of the BP microbial community of M5G were not significantly different from those of UP, they were significantly lower than those of the MP microbial community. The effect of the spatial variation of the alpha-diversity of the pit mud microbial communities on the Baijiu flavor and quality needs to be analyzed in detail by analyzing the metabolic characteristics of bacteria during fermentation and the relationship between the bacteria and flavor components.

In this study, we only sampled at one fermentation time point (60 days after fermentation) and did not analyze the changes in the pit mud microbial community during fermentation. However, previous studies have shown that the microbial community of pit mud changes dynamically during Baijiu fermentation (Zhang, Huang & Hu, 2006; Yu et al., 2021). For instance, Gao et al. (2019) reported that lactobacilli dominated the pit mud microbial community, and the relative abundance of Ruminococcaceae and Clostridiaceae in fermented grains gradually increased with fermentation. Xiang et al. (2013) reported that the fungal *Pichia*, *Saccharomycopsis*, and *Talaromyces* were most abundant in the lees fermented for 1 d, whereas the fungal *Eurotium* and the bacteria *Burkholderia*, *Streptococcus*, and *Lactobacillus* were dominant in the lees fermented for 7 d. Only *Lactobacillus* and *Burkholderia* were prevalent in the lees fermented for 60 days. Therefore, the change in the pit mud microbial community with different liquor-making materials during fermentation needs to be further studied. Simultaneously, the interaction between bacteria and fungi during the fermentation process requires further experimental investigation.

## Conclusions

Firmicutes, Bacteroidetes, Euryarchaeota, Actinobacteria, Synergistetes, Chloroflexi, Chlorobi, Proteobacteria, Tenericutes, and WWE1 dominated the microbial communities in the Luzhou-flavored Baijiu pit muds. The composition of the liquor-making materials significantly affected the composition and spatial distribution of the mud microbial community. The Shannon and Simpson indices of M3G in UP were significantly lower than those in MP and BP, whereas those of M5G in BP were significantly lower than those in MP. T78 of Anaerolinaceae, *Butyrivibrio*, *Dehalobacter_Syntrophobotulus*, *Desulfosporosinus*, *Asteroleplasma*, and vadinCA02 of Synergistaceae were significantly enriched in M3G, whereas *Prevotella*, *Vagococcus*, *Caldicoprobacter*, *Butyrivibrio*, *Coprococcus*, *Dorea*, *Sporanaerobacter*, *Tepidimicrobium*, *TissierellaSoehngenia*, RFN20 of Erysipelotrichaceae, *Sutterella*, 125ds10 of Alteromonadales, *Vibrio*, and *Sphaerochaeta* were significantly enriched in M5G. However, the effects of the composition and spatial distribution differences of pit mud microbial communities on the metabolic characteristics of these bacteria and the effects of the chemical composition and flavor of Luzhou-flavored Baijiu require further study.

## Supplemental Information

10.7717/peerj.12987/supp-1 Supplemental Information 1 Process flow chart introducing the detailed brewing process of Luzhou-flavored Baijiu. The black triangle indicates the phase of sampling.

10.7717/peerj.12987/supp-2 Supplemental Information 2 Principle components analysis profile based on weighted UniFrac distances of pit mud microbial communities.

10.7717/peerj.12987/supp-3 Supplemental Information 3 Partial redundancy analysis results showing the contributions of pit location, and brewing materials to the microbial community difference.

10.7717/peerj.12987/supp-4 Supplemental Information 4 Linear discriminant analysis effect size result comparing pit mud microbial communities between M3G and M5G.

10.7717/peerj.12987/supp-5 Supplemental Information 5 Cladogram plot showing the LEfSe results of pit mud microbial communities among different layers.

10.7717/peerj.12987/supp-6 Supplemental Information 6 LEfSe results of pit mud microbial communities among different layers.

## Additional Information and Declarations

### Data Availability

The following information was supplied regarding data availability: All merged sequences are available in the Genome Sequence Archive database: https://bigd.big.ac.cn/gsa/browse/CRA004711.
