# Supplementary figures and images for: Microbial community spatial structures in Luzhou-flavored liquor pit muds with different brewing materials
Source: PeerJ. 2022 Mar 8;10:e12987. doi: 10.7717/peerj.12987 (PMC8916025; doi:10.7717/peerj.12987)

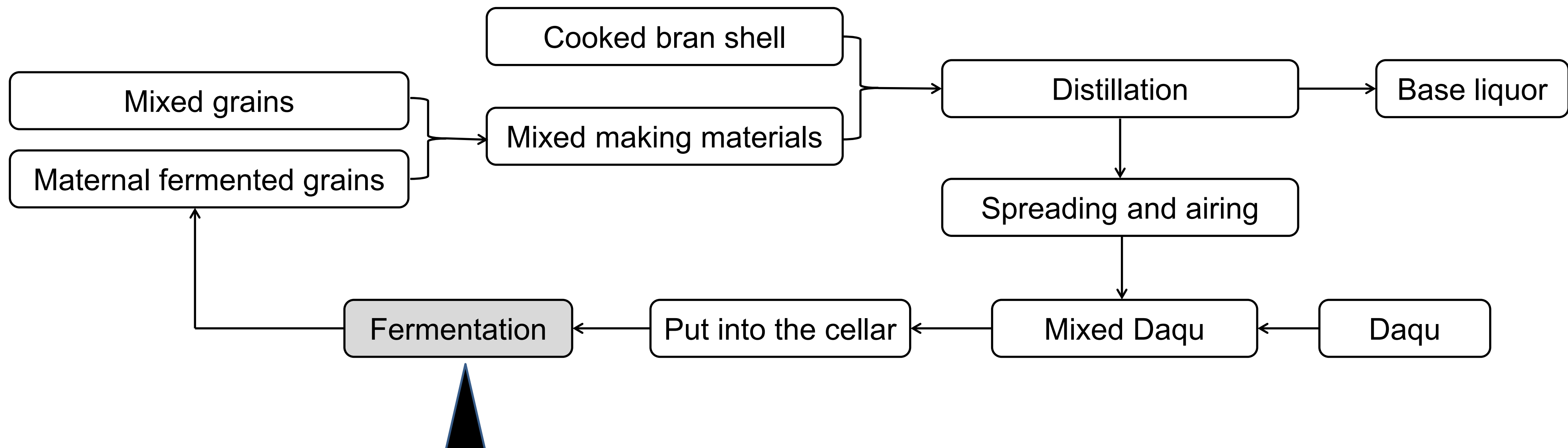

Supplement: Supplemental Information 1 — The black triangle indicates the phase of sampling. [file peerj-10-12987-s001.pdf]

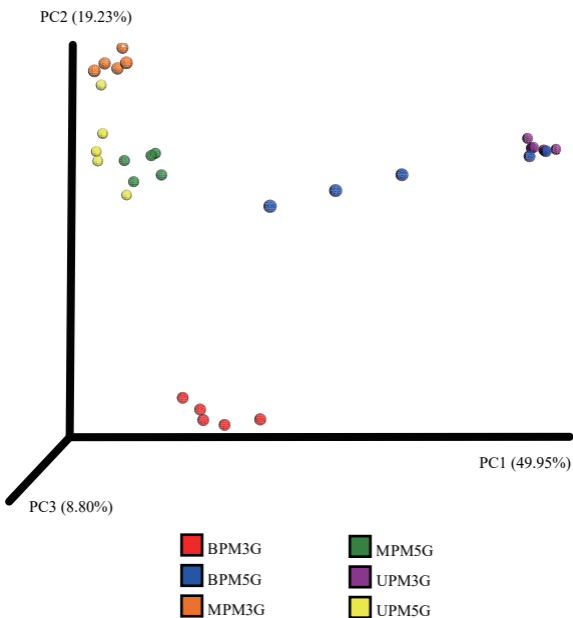

Supplement: Supplemental Information 2 [file peerj-10-12987-s002.pdf]

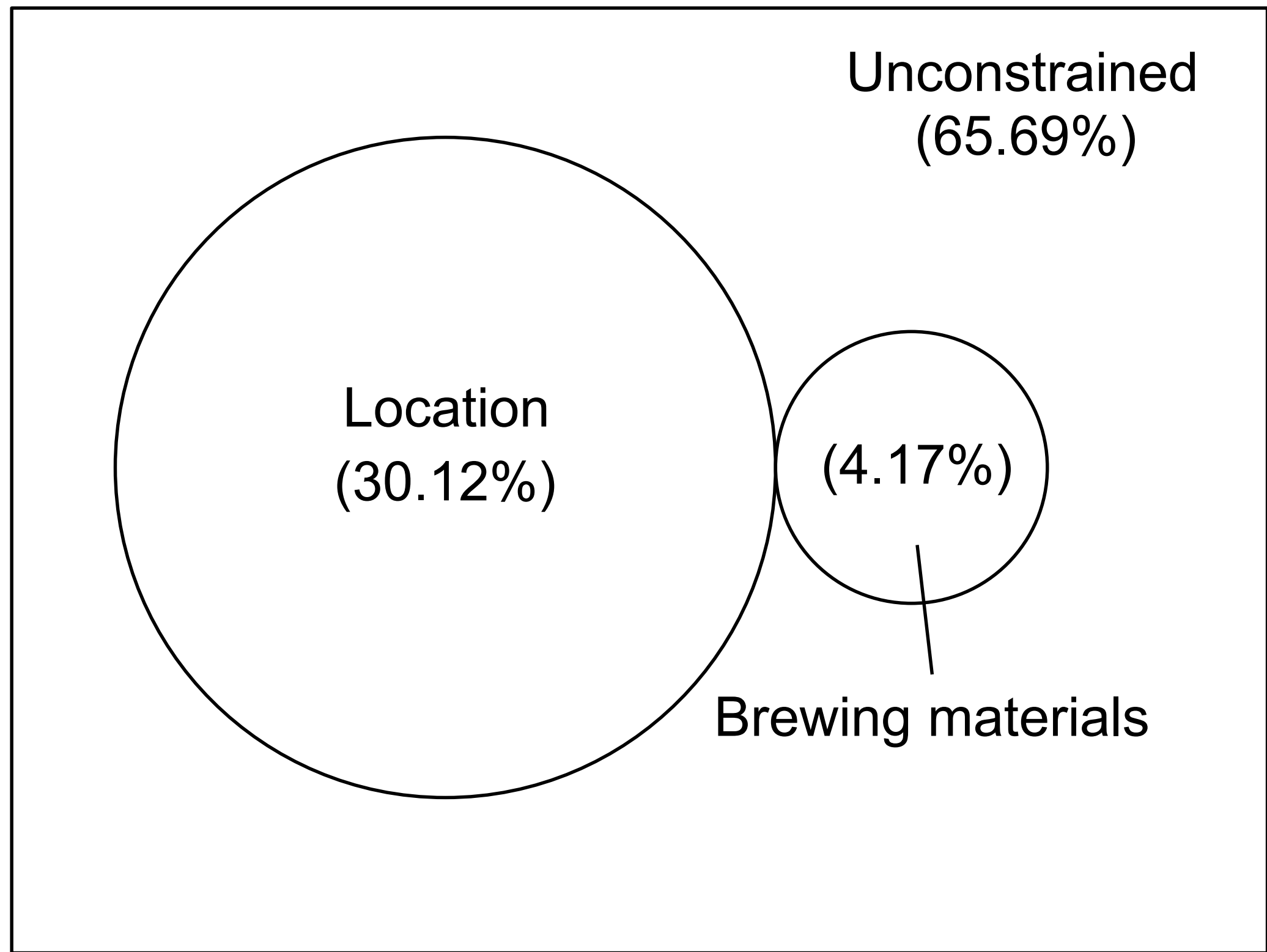

Supplement: Supplemental Information 3 [file peerj-10-12987-s003.pdf]

M3G M5G

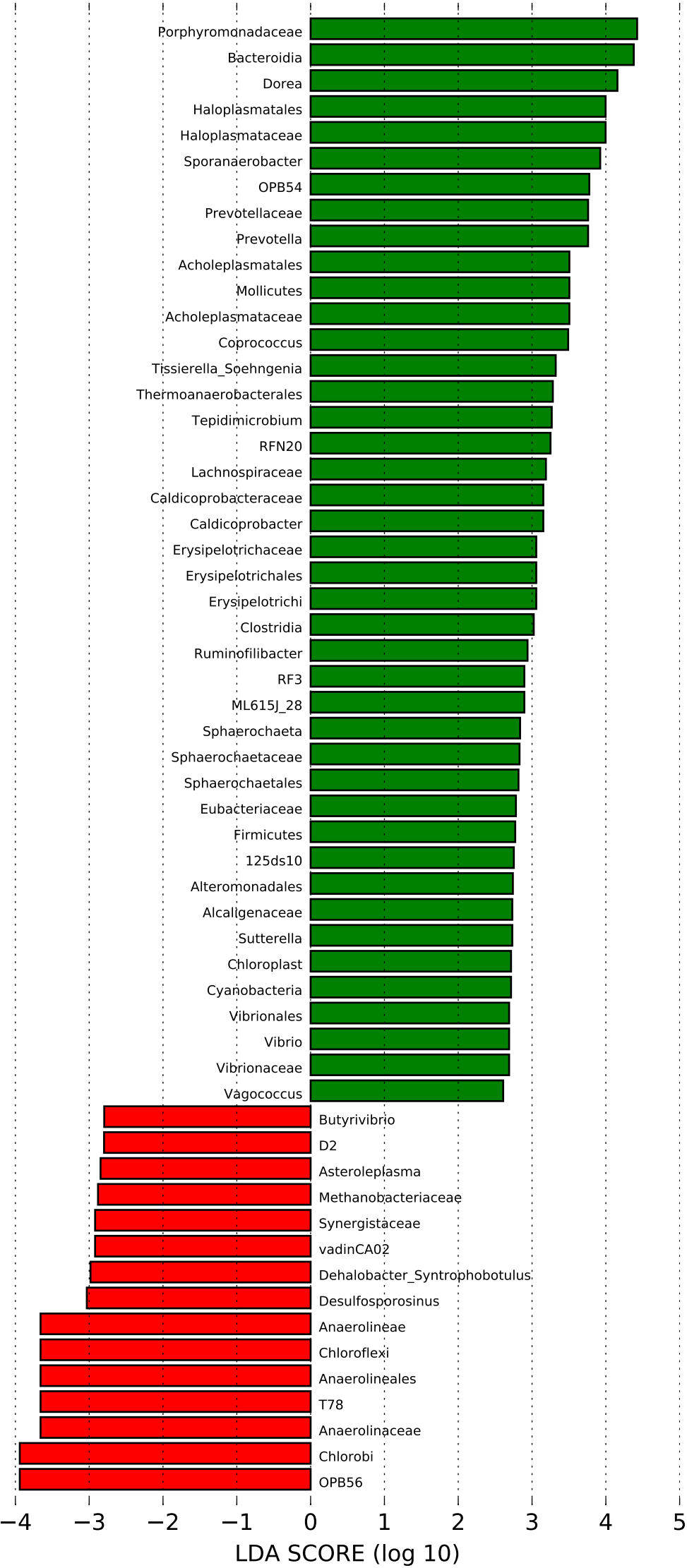

Supplement: Supplemental Information 4 [file peerj-10-12987-s004.pdf]

(A)

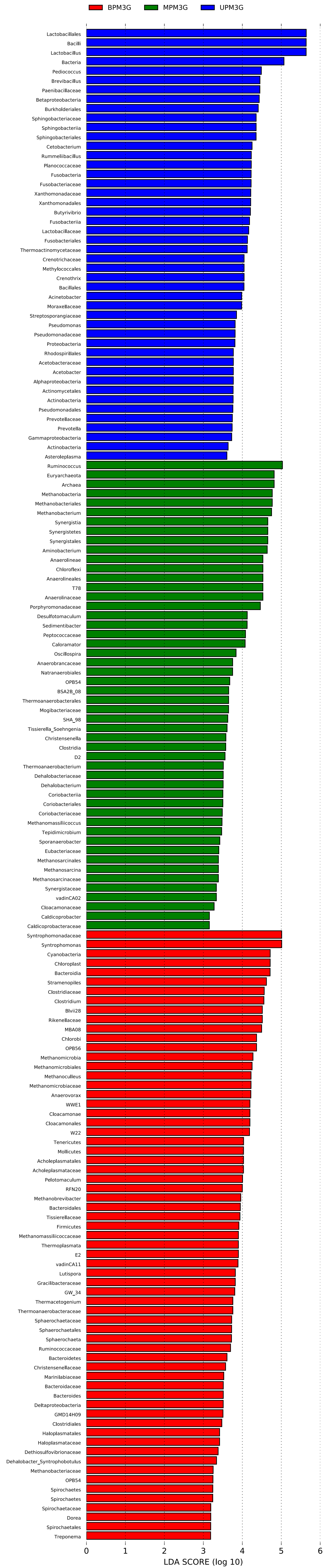

(B)

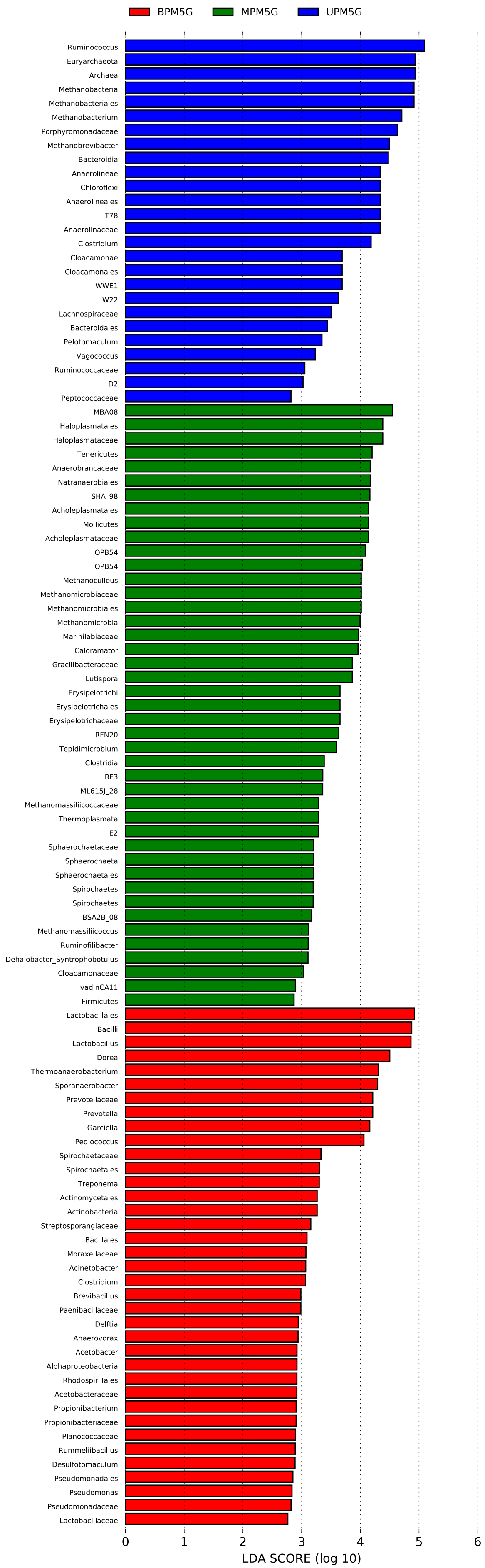

Supplement: Supplemental Information 6 [file peerj-10-12987-s006.pdf]
