# Supplementary material for: Microbial community spatial structures in Luzhou-flavored liquor pit muds with different brewing materials
Source: PeerJ. 2022 Mar 8;10:e12987. doi: 10.7717/peerj.12987 (PMC8916025; doi:10.7717/peerj.12987)
Supplement: Supplemental Information 5 [file peerj-10-12987-s005.pdf]

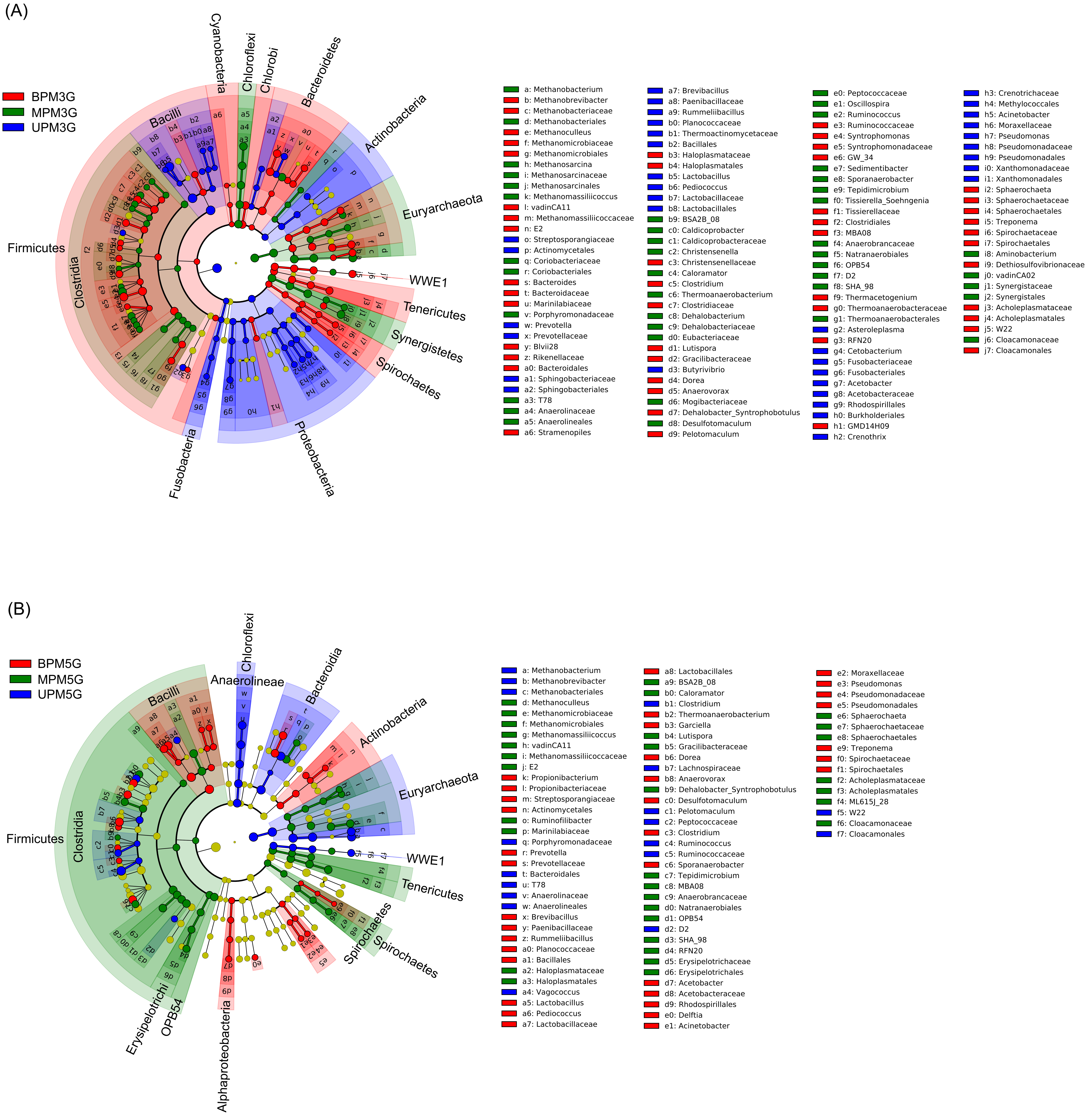
(A)
BPM3G
MPM3G
UPM3G
Cyanobacteria
Chloroflexi
Chlorobi
Bacteroidetes
Actinobacteria
Euryarchaeota
WWE1
Tenericutes
Synergistetes
Spirochaetes
Proteobacteria
Fusobacteria
Firmicutes
Clostridia
Bacilli
a: Methanobacterium
b: Methanobrevibacter
c: Methanobacteriaceae
d: Methanobacteriales
e: Methanoculleus
f: Methanomicrobiaceae
g: Methanomicrobiales
h: Methanosarcina
i: Methanosarcinaceae
j: Methanosarcinales
k: Methanomassiliicoccus
l: vadinCA11
m: Methanomassiliicoccaceae
n: E2
o: Streptosporangiaceae
p: Actinomycetales
q: Coriobacteriaceae
r: Coriobacteriales
s: Bacteroides
t: Bacteroidaceae
u: Marinilabiaceae
v: Porphyromonadaceae
w: Prevotella
x: Prevotellaceae
y: Blvii28
z: Rikenellaceae
a0: Bacteroidales
a1: Sphingobacteriaceae
a2: Sphingobacteriales
a3: T78
a4: Anaerolinaceae
a5: Anaerolineales
a6: Stramenopiles
a7: Brevibacillus
a8: Paenibacillaceae
a9: Rummeliibacillus
b0: Planococcaceae
b1: Thermoactinomycetaceae
b2: Bacillales
b3: Haloplasmataceae
b4: Haloplasmatales
b5: Lactobacillus
b6: Pediococcus
b7: Lactobacillaceae
b8: Lactobacillales
b9: BSA2B_08
c0: Caldicoprobacter
c1: Caldicoprobacteraceae
c2: Christensenella
c3: Christensenellaceae
c4: Caloramator
c5: Clostridium
c6: Thermoanaerobacterium
c7: Clostridiaceae
c8: Dehalobacterium
c9: Dehalobacteriaceae
d0: Eubacteriaceae
d1: Lutispora
d2: Gracilibacteraceae
d3: Butyrivibrio
d4: Dorea
d5: Anaerovorax
d6: Mogibacteriaceae
d7: Dehalobacter_Syntrophobotulus
d8: Desulfotomaculum
d9: Pelotomaculum
e0: Peptococcaceae
e1: Oscillospira
e2: Ruminococcus
e3: Ruminococcaceae
e4: Syntrophomonas
e5: Syntrophomonadaceae
e6: GW_34
e7: Sedimentibacter
e8: Sporanaerobacter
e9: Tepidimicrobium
f0: Tissierella_Soehngenia
f1: Tissierellaceae
f2: Clostridiales
f3: MBA08
f4: Anaerobrancaceae
f5: Natranaerobiales
f6: OPB54
f7: D2
f8: SHA_98
f9: Thermacetogenium
g0: Thermoanaerobacteraceae
g1: Thermoanaerobacterales
g2: Asteroleplasma
g3: RFN20
g4: Cetobacterium
g5: Fusobacteriaceae
g6: Fusobacteriales
g7: Acetobacter
g8: Acetobacteraceae
g9: Rhodospirillales
h0: Burkholderiales
h1: GMD14H09
h2: Crenothrix
h3: Crenotrichaceae
h4: Methylococcales
h5: Acinetobacter
h6: Moraxellaceae
h7: Pseudomonas
h8: Pseudomonadaceae
h9: Pseudomonadales
i0: Xanthomonadaceae
i1: Xanthomonadales
i2: Sphaerochaeta
i3: Sphaerochaetaceae
i4: Sphaerochaetales
i5: Treponema
i6: Spirochaetaceae
i7: Spirochaetales
i8: Aminobacterium
i9: Dethiosulfovibrionaceae
j0: vadinCA02
j1: Synergistaceae
j2: Synergistales
j3: Acholeplasmataceae
j4: Acholeplasmatales
j5: W22
j6: Cloacamonaceae
j7: Cloacamonales
(B)
BPM5G
MPM5G
UPM5G
Chloroflexi
Anaerolineae
Bacteroidia
Actinobacteria
Euryarchaeota
WWE1
Tenericutes
Spirochaetes
Spirochaetes
Alphaproteobacteria
OPB54
Erysipelotrichi
Firmicutes
Clostridia
Bacilli
a: Methanobacterium
b: Methanobrevibacter
c: Methanobacteriales
d: Methanoculleus
e: Methanomicrobiaceae
f: Methanomicrobiales
g: Methanomassiliicoccus
h: vadinCA11
i: Methanomassiliicoccaceae
j: E2
k: Propionibacterium
l: Propionibacteriaceae
m: Streptosporangiaceae
n: Actinomycetales
o: Ruminofilibacter
p: Marinilabiaceae
q: Porphyromonadaceae
r: Prevotella
s: Prevotellaceae
t: Bacteroidales
u: T78
v: Anaerolinaceae
w: Anaerolineales
x: Brevibacillus
y: Paenibacillaceae
z: Rummeliibacillus
a0: Planococcaceae
a1: Bacillales
a2: Haloplasmataceae
a3: Haloplasmatales
a4: Vagococcus
a5: Lactobacillus
a6: Pediococcus
a7: Lactobacillaceae
a8: Lactobacillales
a9: BSA2B_08
b0: Caloramator
b1: Clostridium
b2: Thermoanaerobacterium
b3: Garciella
b4: Lutispora
b5: Gracilibacteraceae
b6: Dorea
b7: Lachnospiraceae
b8: Anaerovorax
b9: Dehalobacter_Syntrophobotulus
c0: Desulfotomaculum
c1: Pelotomaculum
c2: Peptococcaceae
c3: Clostridium
c4: Ruminococcus
c5: Ruminococcaceae
c6: Sporanaerobacter
c7: Tepidimicrobium
c8: MBA08
c9: Anaerobrancaceae
d0: Natranaerobiales
d1: OPB54
d2: D2
d3: SHA_98
d4: RFN20
d5: Erysipelotrichaceae
d6: Erysipelotrichales
d7: Acetobacter
d8: Acetobacteraceae
d9: Rhodospirillales
e0: Delftia
e1: Acinetobacter
e2: Moraxellaceae
e3: Pseudomonas
e4: Pseudomonadaceae
e5: Pseudomonadales
e6: Sphaerochaeta
e7: Sphaerochaetaceae
e8: Sphaerochaetales
e9: Treponema
f0: Spirochaetaceae
f1: Spirochaetales
f2: Acholeplasmataceae
f3: Acholeplasmatales
f4: ML615J_28
f5: W22
f6: Cloacamonaceae
f7: Cloacamonales
